# Digestion of Starch Granules from Maize, Potato and Wheat by Larvae of the the Yellow Mealworm, *Tenebrio molitor* and the Mexican Bean Weevil, *Zabrotes subfasciatus*

**DOI:** 10.1673/031.009.4301

**Published:** 2009-06-19

**Authors:** Elaine A. Meireles, Cíntia N. B. Carneiro, Renato A. DaMatta, Richard I. Samuels, Carlos P. Silva

**Affiliations:** ^1^ Laboratório de Químnica e Função de Proteínas e Peptídeos, Universidade Estadual do Norte Fluminense Darcy Ribeiro, CEP 28013-620, Campos dos Goytacazes, RJ, Brasil; ^2^ Laboratório de Biologia Celular e Tecidual, Universidade Estadual do Norte Fluminense Darcy Ribeiro, CEP 28013-620, Campos dos Goytacazes, RJ, Brasil; ^3^ Laboratório de Entomologia e Fitopatologia, Universidade Estadual do Norte Fluminense Darcy Ribeiro, CEP 28013-620, Campos dos Goytacazes, RJ, Brasil; ^4^ Departamento de Bioquímica, Universidade Federal de Santa Catarina, CEP 88040-900, Florianópolis, Brasil; ^†^ In memoriam

**Keywords:** amylase, microscopy, insect digestion, midgut

## Abstract

Scanning electron microscopy images were taken of starch granules from different sources following exposure *in vivo* and *in vitro* to gut α-amylases isolated from *Tenebrio molitor* L. (Coleoptera: Tenebrionidae) and *Zabrotes subfasciatus* Boheman (Coleoptera: Bruchidae). One α-amylase was isolated from whole larval midguts of *T. molitor* using non-denaturing SDS-PAGE, while two other α-amylase fractions were isolated from whole larval midguts of *Z. subfasciatus* using hydrophobic interaction chromatography., Digested starch granules from larvae fed on maize, potato or wheat were isolated from midgut contents. Combinations of starch granules with isolated α-amylases from both species showed similar patterns of granule degradation. *In vitro* enzymatic degradation of maize starch granules by the three different α-amylase fractions began by creating small holes and crater-like areas on the surface of the granules. Over time, these holes increased in number and area resulting in extensive degradation of the granule structure. Granules from potato did not show formation of pits and craters on their surface, but presented extensive erosion in their interior. For all types of starch, as soon as the interior of the starch granule was reached, the inner layers of amylose and amylopectin were differentially hydrolyzed, resulting in a striated pattern. These data support the hypothesis that the pattern of starch degradation depends more on the granule type than on the α-amylase involved.

## Introduction

Starch is a major constituent of seeds and grains that is used as a source of fuel and carbon for germinating seeds but it is also used as nutrient source by granivorous insects. In order to understand the adaptability of insect pests to their food sources, qualitative and quantitative determination of digestive parameters must be carried out. A complete description of the digestive process of starch granules involves the quantification and spatial distribution of enzymes involved in digestion of carbohydrates (endo- and exocarbohydrases). However, starch granules present a special difficulty in terms of digestion, when compared to other nutrients such as proteins (Baker and Woo 1992) because they are synthesized as granules that are intrinsically resistant to degradation in their native or raw state (Buléon et al. 1998; Gallant et al. 1997). Taking this information into account is essential in a full description of the process by which insects exploit this important carbohydrate source.

In spite of the importance of starch utilization by insects, the process of starch granule digestion has received relatively little attention, compared to the greater number of studies that used refined starch, rather than “unaltered” or granular starch. Raw starch granules are natural substrates, and two articles from the beginning of the last decade are the pivotal works that utilized starch granules to assay for α-amylase activity (Baker and Woo 1992; Baker et al. 1992) followed by others, as for example, the characterization of the α-amylase in *Bemisia tabaci* (Gennadius) (Homoptera: Aleyrodidae) (Cohen and Hendrix, 1994). These authors have demonstrated that the mechanical damage caused by mastication is crucial to the digestive process of starch granules. The specificity of α-amylase to plant α-amylases inhibitors can also be different when starch granules are used as substrates, as compared to gelatinized starch (Silva et al. 2001b). For most insect species studied, information on the digestion of raw starch is very scarce. In contrast, the kinetic aspects of the α-amylases involved in this process have been well studied (Baker and Woo 1992; Silva et al. 1999; Silva et al. 2001a, Silva et al. 2001b; Terra and Ferreira 2005). To better understand the process by which larvae of granivorous insect pests deal with different starch granules, we investigated the *in vivo* and *in vitro* digestion of raw starch granules from wheat, maize and potato by two insect species, the yellow mealworm, *Tenebrio molitor* L. (Coleoptera: Tenebrionidae) and the Mexican bean weevil, *Zabrotes subfasciatus* Boheman (Coleoptera: Bruchidae), both of which are serious pests of stored grains and pulses. The results showed that the overall patterns of starch granule digestion depends more on granule type than on the α-amylasess involved in the process.

## Materials and Methods

### Insect rearing

The colony of the *Z. subfasciatus* was supplied originally by Prof. F.M. Wiendl of the Centro de Energia Nuclear na Agricultura, Piracicaba, São Paulo, Brazil. A stock culture of this species has been maintained since 1994. The insects were reared on black-eye pea, *Vigna unguiculata* (L.) Walp. (Fabales: Fabaceae), seeds in darkness and maintained at 29 ± 1° C and 65 ± 5 % RH. To have a source of inducible α-amylasess, seeds of the common bean, *Phaseolus vulgaris* L. (Fabales: Fabaceae) were also used to feed *Z. subfasciatus* (Silva et al. 2001b). The inducible α-amylases are expressed when the larvae of *Z. subfasciatus* are fed on the α-amylase inhibitor-containing seeds of *P. vulgaris*. A stock culture of *T. molitor* has been maintained since 2002 under natural photoperiod conditions on wheat bran at 29 ± 1° C and 65 ± 5 % RH.

### Preparation of samples from insects

Final instar larvae were cold immobilized and dissected to remove the whole midgut in cold 250 mM NaCl. Only larvae with food-filled guts were chosen for dissection. After removal of the whole gut, the adhering unwanted tissues were removed and the pooled midguts, unless otherwise stated, were homogenized in cold distilled water using a hand-held Potter—Elvehjem homogenizer immersed in ice. Midgut tissue homogenates were centrifuged at 20,000 × g for 30 min at 4° C and the supernatants were collected and used as enzyme sources.

Partially digested starch granules were also collected from larval midgut contents. Midguts were divided into anterior, middle and posterior parts. The anterior and posterior parts were discarded, while the medial part was split open along its length and gently pressed to drive out the contents into the surrounding distilled water. The suspensions were collected with the aid of a fine capillary and centrifuged at 600 g for 5 min. The pellets were washed two times with distilled water and dried at 40 °C. Alternatively, they were suspended in 95 % ethanol for examination by scanning electron microscopy.

### Feeding the larvae of *T. molitor* and *Z. subfasciatus*

Starch granule digestion by larval *T. molitor* and *Z. subfasciatus* was studied by feeding larvae commercial starch (Sigma®) of maize (*Zea mays*), potato (*Solanum tuberosum*) or wheat (*Triticum vulgaris*). To feed *Z. subfasciatus* larvae, starch granule flour was inserted and compacted inside gelatin capsules. Two fourth instar larvae were transferred into a cavity made in the compacted mass of flour in one half of a gelatin capsule. Feeding larvae were maintained in the capsules for 48 h (Silva et al. 1999). Larval *T. molitor* feeding was carried out by immersing last instar larvae in starch flour for 48 h. After the feeding period, larvae of both species were removed and the midgut was dissected for observation of the ingested granules or for preparation of midgut homogenates.

### Conditions for assay of hydrolases

α-Amylase activity was determined, unless otherwise stated, by using a 3,5-dinitrosalicylic acid reagent prepared according to Noelting and Bernfeld (1948). In the case of gelatinized starch, sources of enzymes (25 µl) were incubated with 25 µl substrate/buffer solution (1% potato soluble starch in 100 mM acetate buffer pH 5.5 containing 4 mM CaCl_2_ and 40 mM NaCl). Both *T. molitor* and *Z. subfasciatus* have high amylase activity at pH 5.5. The assay was terminated by the addition of 200 µl 3,5-dinitrosalicylic acid. The solution was heated in a boiling water bath for 5 min, cooled, and after the addition of 200 µl of distilled water, the absorbance was read at 550 nm. All assays were performed at 30°C. Incubations were carried out for at least four different periods of time, unless otherwise stated, and the initial rates of hydrolysis were calculated. One enzyme unit was expressed as the quantity of enzyme that produces 1 µmol of maltose equivalent per minute.

*In vitro* digestion of starch granules was performed by incubating 200 µl of 2 % (w/v) granule suspensions with an equal volume of the enzyme sources (0.3 U α-amylases) in a reaction mixture containing 50 mM acetate buffer (pH 5.5), 0.2% NaN_3_, 10 µM E-64, 5 µg/ml pepstatin A, 20 mM NaCl and 2 mM CaCl_2_. The reactions were conducted at 30° C for different periods of time. The suspensions were centrifuged at 7000 g for 5 min and divided into supernatants and packed starch granules. The granules were suspended and washed twice in 2.0 ml of water and finally suspended in 95% ethanol for scanning electron microscopy.

### *In gel* assays

Midgut a—amylases were detected using in-gel assays following non-denaturing SDS-PAGE (Campos et al. 1989; Silva et al. 1999). Chromatographic fractions were diluted two-fold in sample buffer [2.1 ml distilled water + 0.5 ml 0.5 M Tris—HCl, pH 6.8 + 0.4 ml glycerol + 0.8 ml 10% SDS (w/v) + 0.2 ml 1% bromphenol blue (w/v)] (note the absence of 2-mercaptoethanol) and subjected to electrophoresis without boiling the samples, using minigels (10 cm × 7 cm × 1 mm) in a BioRad (www.biorad.com) Mini Protean 3 apparatus. Proteins were separated on 7.5% (samples from *Z. subfasciatus*) or 12% (samples from *T. molitor*) acrylamide resolving gel and 6% acrylamide stacking gel. The runs were carried out at 4°C and 150 V using pre-cooled buffers. After the runs, gels were transferred to 2.5% Triton X-100 (w/v) for 20 min at room temperature, and then transferred to a substrate/buffer solution [1% gelatinized potato starch (w/v), 100 mM acetate 20 mM NaCl — 2 mM CaCl_2_, pH 5.5] and incubated at 30°C for 30 min. After briefly rinsing the gel in water, amylolytic activity was stopped by transferring the gels to the staining solution [1.3% I_2_ (w/v), 3% KI (w/v)]. After staining, light bands against the dark background indicated the presence of active α-amylases.

### Isolation of α-amylases from larval *T. molitor* and *Z. subfasciatus*

α-Amylase from larval *T. molitor* was obtained by elution following semi-denaturing electrophoresis as described above. Induced and constitutive α-amylases from larval *Z. subfasciatus* were obtained by hydrophobic interaction chromatography on a phenyl-agarose column essentially as described by Silva et al. (2001a).

Samples of 50 larval *Z. subfasciatus* midguts were homogenized in an aqueous solution containing 10 µM E-64 and 5 µg/ml pepstatin A using a Potter—Elvehjem homogenizer, and then centrifuged at 20,000 g, 30 min, 4°C. The supernatant was adjusted to 1 M with ammonium sulfate, and was then applied to a phenyl—agarose column (10 × 0.5 cm id) equilibrated with 10 mM imidazole buffer, pH 6.0 containing 1 M ammonium sulfate, using an Econo System (BioRad). The column was washed with 10 ml of equilibration buffer and eluted with a 40 ml linear gradient decreasing to 0 M ammonium sulfate in imidazole buffer, followed by a 10 ml isocratic elution using imidazole buffer without ammonium sulfate. The flow rate was 1.0 ml/min and 1.0 ml fractions were collected and placed on ice immediately.

The broad α-amylases activity peak that eluted between 300 mM—0 M ammonium sulfate was collected and pooled and then subjected to another chromatographic step on the same column, but using a stepwise elution procedure. The column was equilibrated with 10 mM imidazole buffer, pH 6.0 containing 1 M ammonium sulfate. The enzyme fraction was adjusted to 1 M with ammonium sulfate, it was applied to the column and washed with 5 ml of equilibration buffer, followed by 20 ml of a linear gradient of 1 M - 300 mM ammonium sulfate in imidazole buffer, followed by 20 ml of 300 mM ammonium sulfate in imidazole buffer, followed by a linear gradient of 5 ml of 300 mM - 0 M ammonium sulfate in imidazole buffer, and finally 25 ml of imidazole buffer only. The flow rate was 1.0 ml/min and fractions of 1.0 ml were collected and placed on ice immediately. Runs were performed at room temperature. Recovery of enzyme activity was 80–100%.

### Scanning electron microscopy analysis of starch digestion

Scanning electron microscopy images were obtained of starch granules subjected to hydrolysis by the different amylase fractions and of starch granules obtained from insect intestinal lumens. Starch granule preparations were suspended in 95% ethanol and applied to a specimen stub. Samples were then coated with gold and observed using a Zeiss 964 Scanning Electron Microscope (www.zeiss.com) at 15 kV.

## Results and Discussion

**Figure 1.**
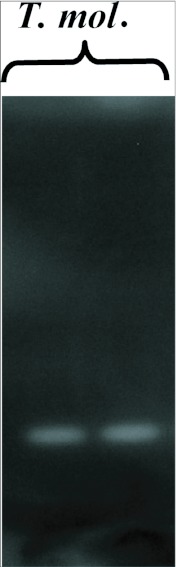
α-Amylase from larvae of *Tenebrio molitor* resolved by non-denaturing SDS-PAGE.

**Figure 2.**
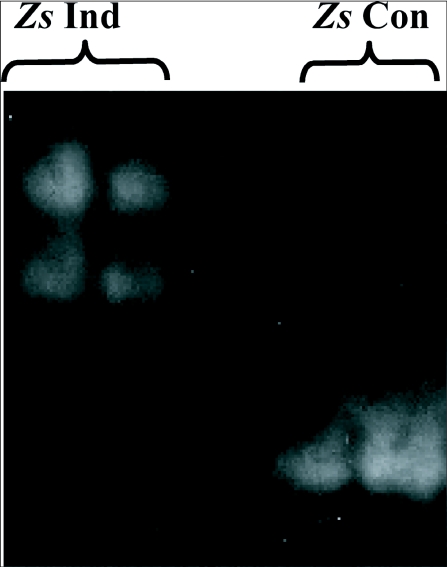
Mildly-denaturing SDS—PAGE followed by *in gel* assay of α—amylases from larvae of *Zabrotes susbfasciatus*. Samples from the *Z. subfasciatus* amylase fractions obtained in the hydrophobic interaction chromatography were run on SDS–7.5% polyacrylamide gels. After a renaturation step, α—amylase activities were assayed as detailed in the text. *Zs* Ind: inducible α-amylases from *Z. subfasciatus*; *Zs* Con: constitutive α-amylase from *Z. subfasciatus*. Larvae of *Z. subfasciatus* express a major constitutive α-amylase when feeding on α-amylase inhibitor-free seeds, but express two slow migrating α-amylase isoforms, when fed on amylase inhibitor-containing diets (see Silva et al. 2001a,b).

**Figure 3.**
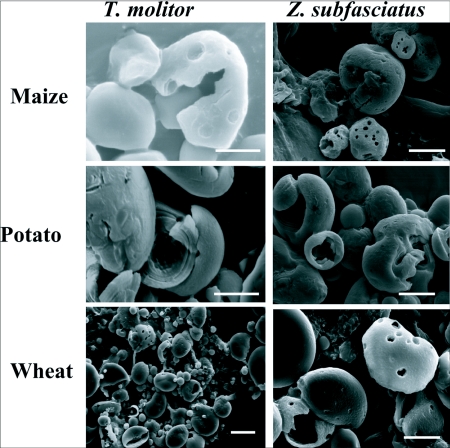
Scanning electron micrography (SEM) of starch granules during *in vivo* digestion. Final instar larvae of *Tenebrio molitor* and *Zabrotes subfasciatus* were fed starch flour for 48 h. After this feeding period, midguts were dissected and starch granules were isolated, washed, dehydrated and observed using SEM. Bars: 5 mm.

**Figure 4.**
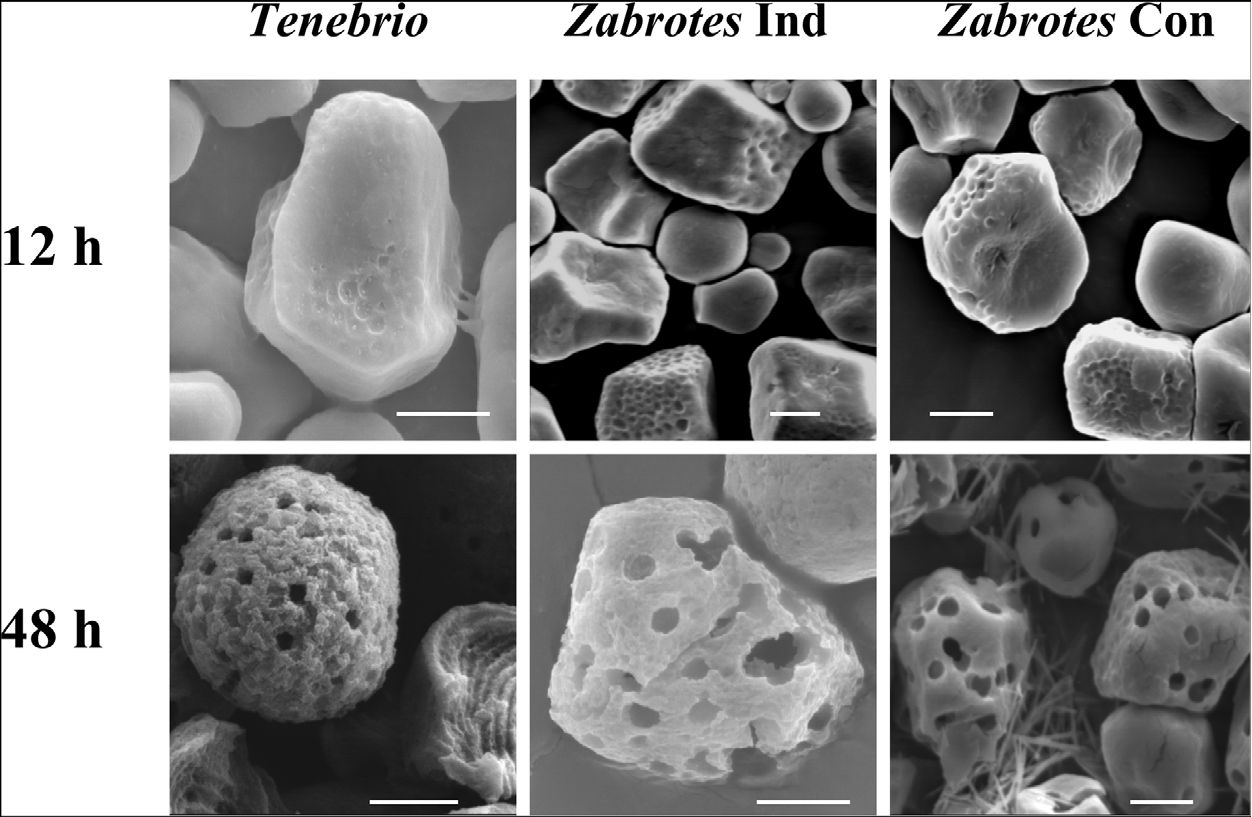
Scanning electron micrographs of starch granules from maize during *in vitro* digestion by *Tenebrio molitor* amylase and by two amylase fractions purified from larval *Zabrotes subfasciatus* (see Figs. 1 and 2). Starch granules were incubated with 0.5 U of the α-amylases for 12 h and 48 h. After incubation, degraded starch granules were washed, dehydrated and observed. Note the formation of pits and crater-like holes on the surface of the granules after 12 h, and that these holes increased in number after 48 h of incubation, leaving a “Swiss cheese” pattern of the digested starch granules. Bars: 5 mm.

One α-amylase fraction was isolated from whole midgut of larval *T. molitor* following non-denaturing electrophoresis (Figure 1). Two α-amylase fractions were obtained from larval midgut homogenates of *Z. subfasciatus* fed on *P. vulgaris* using two sequential hydrophobic interaction chromatography steps (Figure 2). Scanning electron microscopy images were obtained of native starch granules that had been hydrolyzed *in vivo* within the gut, and *in vitro* with different α-amylase fractions. All combinations of starch granules with different enzyme sources resulted in similar patterns of granule degradation (Figures 3 and 4). The internal parts of the granules were more highly degraded than the granule surface, irrespective of the granule origin or amylase source. The fragments of the granules undergoing *in vivo* digestion seemed to have greater erosion in the inner core, leaving external parts with holes, when the larvae had been fed maize and wheat starch granules, and an apparently undamaged surface when they were fed potato starch granules (Figures 3 and 4). Moreover, some internal parts of the starch granules were less digested than other parts, exposing the lamellar organization of the granules.

Analysis of the images showed that the patterns of digestion of the different starch granules were similar, even when digested by different α-amylases (Figures 3 and 4). *In vivo* degradation of maize, potato, and wheat starch granules was similar irrespective of the insect (Figure 3). *In vitro* enzymatic degradation of maize starch granules by the three different insect α-amylases began by creating small holes and crater-like areas on the surface of the granules (Figure 4). Over time, holes increased in number and area, resulting in extensive degradation of the granule structure. Digestion of granules from potato did not show formation of pits and craters on their surface, but had extensive interior erosion (Figure 3). Similar results were obtained for bacterial and plant α-amylases (Helbert et al. 1996; Gallant et al. 1997; Sarikaya et al. 2000; Smith et al. 2005). This same pattern of granule degradation was also observed during *in vitro* and *in vivo* digestion of wheat starch granules by larval *Tribolium castaneum* α-amylases (Baker et al. 1992). A comparison of the digestion of starch granules isolated from two legume species, *V. unguiculata* and *P. vulgaris*, also showed that different α-amylases produced the same pattern of granule degradation, but with liberation of different dextrins (Silva et al. 2001a). For all starch granules studied, as soon as the interior was reached, the inner layers of amylose and amylopectin were differentially hydrolyzed, resulting in a striated pattern (Figures 3 and 4 in this study; Baker et al. 1992; Silva et al. 2001a). Starch granules from maize and wheat showed a similar pattern of *in vitro* degradation by plant amylases, characterized by the “Swiss cheese” pattern of the digested starch granules (Sarikaya et al. 2000).

Undamaged raw starch granules can be very resistant to digestion (Baker et al. 1992; Gallant et al. 1997; Silva et al. 2001a). Therefore, mastication may be a crucial step in their utilization by insects. The internal layers of the granules are more susceptible to digestion than their surfaces (Figures 3 and 4). As the granules are synthesized from inside to outside layers (Buléon et al. 1998), the more susceptible core is protected by the resistant external layers, conferring resistance to digestion, but the chewing process permits access to the more susceptible parts of the granules. These facts explain why *in vitro* digestion is very much slower than the *in vivo* process. Therefore, exposure of the internal parts by the mastication process is crucial for digestion of starch granules that have a resistant surface, as is the case for potato starch granules.
